# Dr. George Clark

**Published:** 1911-10

**Authors:** 


Dr. George Clark of Byron, was killed by the overturning of his automobile, September 22, 1911. He was 35 years old.

